# Satisfaction and wellbeing of general surgery trainees in the Saudi Arabian residency educational environment: A mixed-methods study

**DOI:** 10.1016/j.sopen.2024.06.011

**Published:** 2024-07-05

**Authors:** Mohammed F. Shaheen, Abdulrahman Y. Alhabeeb, Moustafa S. Alhamadh, Meshal A. Alothri, Rakan S. Aldusari

**Affiliations:** a College of Medicine, King Abdullah International Medical Research Center, King Saud bin Abdulaziz University for Health Science, Riyadh, Saudi Arabia; b Organ Transplant Center and Hepatobiliary Sciences Department, King Abdulaziz Medical City, Ministry of the National Guard - Health Affairs, Riyadh, Saudi Arabia

**Keywords:** Surgical education, General surgery, Residency program, Satisfaction, Burnout

## Abstract

**Background:**

Surgical residency training is prominently demanding and stressful. This can affect the residents' wellbeing, work-life balance and increase the rates of burnout. We aimed to assess rates of satisfaction and burn-out among GS residents in the national training programs and provide a subsequent in-depth analysis of the potential reasons.

**Method:**

A sequential explanatory mixed-methods study was conducted using an online survey and virtual interviews. The validated abbreviated Maslach Burnout Inventory (aMBI) was used to assess burnout while satisfaction was assessed via 5-points Likert scale.

**Results:**

After excluding incomplete responses from the total 74 received, 53 were analyzed. The average participant age was 27.4 ± 2 years, with females comprising 52 % of the sample. Junior residents made up 58.5 %, and nearly half −45 %- considered quitting GS training. Moderate to high burnout rates were noted on each aMBI subscale, ranging from 41.7 % to 62.5 %. The majority of residents expressed dissatisfaction with the level of research engagement (81.1 %), supervision, and mentorship. However, operative exposure was a source of satisfaction. Dissatisfaction rates with intra-operative learning, academia, teaching, and clinical exposure were 62.3 %, 52.8 %, 50.9 %, and 35.8 %, respectively. Interviews revealed surgical case flow and a friendly work environment as major satisfaction sources. Conversely, lack of academic supervision and suboptimal hands-on training were major dissatisfaction sources.

**Conclusion:**

Dissatisfaction and burn-out is prevalent among national GS training programs. Sub-optimal educational delivery and low-quality hands-on operative exposure -rather than lack of exposure to cases- seem to be the culprit.

## Introduction

The educational environment (EE) encompasses all factors that affect the educational process of learners, including the physical environment, emotional climate, and intellectual atmosphere [1,2]. The attributes of the EE have a significant impact on the learning practices and outcomes of residency training programs [3]. Therefore, it is crucial for residency programs to evaluate their EE and obtain insights from their residents to ensure the quality of educational conduct, improve trainees' wellness, and identify areas for improvement [[4], [5], [6]].

In Saudi Arabia, the Saudi Commission for Health Specialties (SCFHS) is responsible for overseeing residency training programs. The SCFHS established and accredited the five-year general surgery (GS) training program in multiple hospitals in 2002, with ongoing improvements being made to enhance residents' educational experiences [7]. However, studies evaluating residents' satisfaction with the surgical programs' EE in Saudi Arabia are scarce and reflect low levels of satisfaction [8,9]. On the other hand, in USA, general surgery residents reported significantly higher levels of satisfaction with their training programs, reaching as high as 85.2 % [10].The key factors that influence the satisfaction levels of residents in the general surgery training program in Saudi Arabia remain nebulous. Understanding these factors is crucial to develop strategies that can improve these satisfaction levels, potentially aligning them with the high satisfaction rates observed in similar programs.

The purpose of this mixed-methods study is to gain an inclusive understanding of the elements of the EE that affect general surgery trainees the most. Quantitative measures of residents' satisfaction and wellbeing were followed by semi-structured interviews to gain in-depth insights into their experiences. The study aims to provide recommendations for improving the EE and positively influencing the wellbeing of surgical trainees.

## Methods

The study utilized an explanatory sequential mixed-methods design with an online survey sent to general surgery residents in all 38 training hospitals operating under the umbrella of the SCFHS general surgery training program. The study obtained IRB approval from King Abdullah International Medical Research Center (study number: RC201445/R) and conducted between December-2020 to January-2022.

The on-line survey comprised 26 questions on demographics, satisfaction with various elements of the program's EE [11], and burnout using the Abbreviated Maslach Burnout Inventory (aMBI). The aMBI scoring was performed and stratified following the cutoffs scores previously identified in the literature [12]. Cutoff scores for high risk of burnout in each subscale were ≥ 11 for emotional exhaustion, ≥7 for depersonalization and ≤ 12 for personal accomplishment [13]. Participants who provided complete responses to either the satisfaction or burnout scales were included.

In order to gain in-depth insights into residents experiences, an optional request to be interviewed was also included. Semi-structured interviews were conducted with residents who volunteered and explored the elements of the EE and wellbeing. The conducted semi-structured interviews explored various elements of the EE and wellbeing. The questions covered 5 domains, namely, 1- teaching and learning, 2- work environment and operative experience, 3- interpersonal relationships and quality of life, 4-mentorship, and research. The interviews were conducted virtually through Microsoft teams. All interviewees were guaranteed anonymity, and no identifying information was used. All interviews were converted to verbatim transcripts.

Quantitative data were analyzed using SPSS v.27 to yield descriptive statistics, *t*-test, and Pearson correlation, while qualitative data underwent line-by-line initial coding by three independent reviewers followed by searching for themes and then defining them in thematic analysis using Dedoose on-line Software.

## Results

**Fig. 1:**
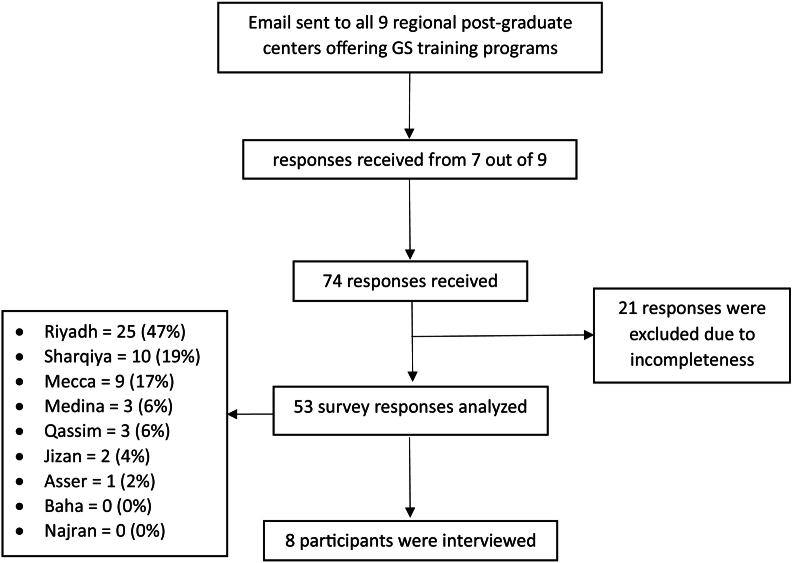
Survey response summary.

**Fig. 2:**
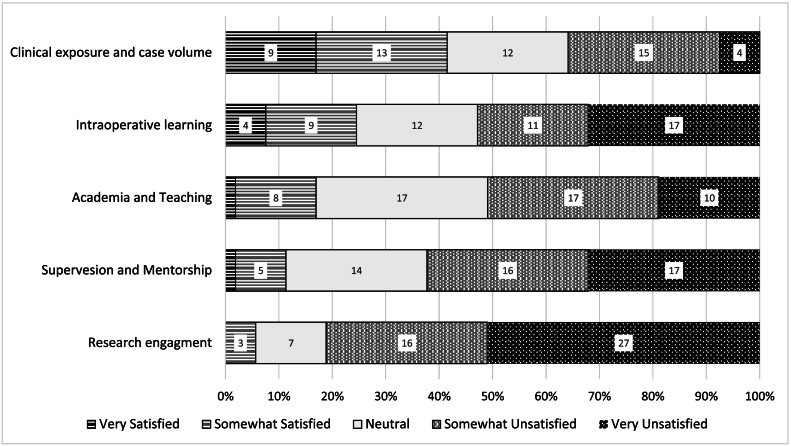
Trainees' satisfaction with different elements of the educational environment.

Of the 74 survey responses received from 7 of the 9 training regions, 53 were analyzed after exclusion of deficient responses. Based on the estimated reach of 270 to 300 general surgery residents in Saudi Arabia through the method implied, the estimated response rate is approximately between 25 % and 27 % [14,15]. The majority of responses were from programs in Riyadh, Sharqiya, and Mecca regions, which harbors 27 out of the 38 training hospitals (Fig. 1). The mean age of participants was 27.4 ± 2 years, with even gender distribution. Junior residents accounted for 58.5 % of responses. Half of the participants reported being unsatisfied with the training program (Table 1). Most residents reported dissatisfaction with research, mentorship, academia, and intraoperative teaching ranging from 50.9 % to 81.1 % of responses, while 41.5 % of responses reported their satisfaction with case mix and volume (Fig. 2). Nearly half of the residents considered changing the general surgery program, with loss of work-life balance and unfriendly work environment being the primary quoted reasons. The overall level of satisfaction – measured on a 5-point Likert scale (1: very dissatisfied, and 5: very satisfied) – was 2.4 ± 1.

**Table 1:** Demographics data:

Demographics	
**Age,** mean ± SD	27.4 ± 2 *years*
Female gender, *n (%)*	27 (52 %)
Marital status, *n (%)*	
- Single	35 (66 %)
- Married	13 (25 %)
- Separated/divorced	5 (9 %)
Postgraduate year (PGY) of training, *n (%)*	
- PGY 1	20 (38 %)
- PGY 2	11 (21 %)
- PGY 3	11 (21 %)
- PGY 4	6 (11 %)
- PGY 5	5 (9 %)
Satisfaction with the training program	
Overall Satisfaction with the GS training program, *n (%)*	
- Not satisfied at all	12 (23 %)
- Not satisfied	15 (28 %)
- Neutral	16 (30 %)
- Satisfied	10 (19 %)
- Very satisfied	0 (0)
Consider quitting the program?, *n (%)*	
- Yes	24 (45 %)
Reasons for considering to quit?, *n (%)*	
- Loss of work-life balance	15 (60 %)
- Unfriendly work environment	15 (60 %)
- Difficulty of surgical training	9 (38 %)
- Lack of hands-on training	4 (17 %)
- Better opportunity in another specialty	4 (17 %)
- Family or spousal related factors	3 (13 %)
- Financial burden	2 (8 %)
- Lost interest	1 (4 %)

**Fig. 3:**
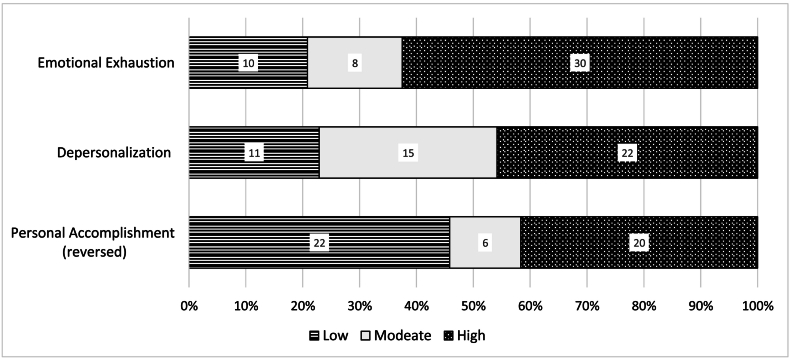
Burnout survey (aMBI) results.

Burnout symptoms screening through aMBI reflected moderate to high levels of emotional exhaustion and depersonalization with moderate levels of personal accomplishment (Fig. 3). The average score for personal accomplishment was 13.1 ± 3.3, emotional exhaustion 11.7 ± 5.1, and depersonalization 7.1 ± 4.8. Low score for personal accomplishment was noted in 41.7 % of participants, while high scores for emotional exhaustion and depersonalization was noted in 62.5 % and 45.8 %, respectively. High burnout risk has been defined as the co-presence of high scores in the emotional exhaustion and depersonalization subscales which occurred in 37.5 % of the cohort. Overall satisfaction with the training program significantly correlated with low reported levels of emotional exhaustions; *r* = −0.42, *p* = 0.003. Additionally, residents who reported the desire to quit, had significantly lower satisfaction scores for clinical cases volumes; 2.7 versus 3.5, *p* = 0.02, and intraoperative teaching; 1.9 versus 3.1 *p* < 0.001.

Eight residents voluntarily signed up for a subsequent semi-structured interview and all were interviewed. Five of the interview participants were males and half of them were from the central region while the others were from the eastern and western regions. Half of the participants were satisfied with their training program. Each interview lasted an average of 35 min as they ranged from 21 min to 64 min. Qualitative analysis of the transcripts revealed several key themes that either enhanced or detracted from satisfaction. Additionally, several key elements of the EE seem to be associated with the overall participant's sense of satisfaction or dissatisfaction (Table 2).

**Table 2:** Differences in reported experiences of interviewees based on their satisfaction with their GS training program.

		Stratified Trainee's Description During Interviews			
		Coded theme	Coded subtheme	Satisfied interviewee *(n out of 4)*	Dissatisfied interviewee *(n out of 4)*
Educational Environment Elements	Physical Environment	Surgical Cases mix and flow	Optimal	4	2
			Insufficient	0	1
		Simulation/Hands-on training material	Available/Utilized	4	0
			Not available/Not Utilized	0	2
	Learning content and format	Academic activities	Well-structured	1	0
			Poorly supervised	2	2
		Bed-side teaching	insufficient	3	2
		Intraoperative teaching and skill learning	Optimal	2	1
			insufficient	2	3
		Research opportunities and engagement	Available	2	0
			Not available	1	4
	Roles and Responsibilities	Patient care	Appropriate team integration	1	0
			Clerkship role	2	3
			Insignificant role	0	1
		Operative role	Hands-on/effective assistant	3	1
			Observer/non effective assistant	1	3
	Learner wellness	Subjective feeling	Happy	1	1
			Unhappy	2	2
			Symptoms of burn-out	1	1
		Work- life balance	Reasonable balance	0	3
			Long working hours	2	1
	Social Environment	Mentor/supervisor relations	Good and established	2	0
			Poor or not established	0	2
		Work environment	Friendly	2	1
			Hostile	2	2
		Peer relations	Good	3	1
			Poor	1	3

The Sources of satisfaction that was reiterated the most in the interviews were, presence of good flow of surgical cases, graded operative autonomy, supportive mentorship, friendly work culture related to seniors, peers, and hospital staff. While the sources leading to dissatisfaction were, inadequate senior supervision and feedback for academic activities, bed-side assessment and decision making as well as noneffective operative and nonoperative roles leading to suboptimal hands-on training and a predominantly clerking responsibility. Reasons quoted by the interviewee explaining these factors were the presence of junior physician in non-training positions, private practice for senior surgeons and medicolegal worries and restrains. Availability of research opportunities to trainees and the work-life balance were also mentioned as factors affecting the level of satisfaction but were less prominent. Since the study period was close to the time of lock down related to COVID-19 pandemic, two of the interviewees mentioned that it affected their experience negatively. Additionally, the ongoing oversight over training programs by the SCFHS were deemed a source of optimism for improvement by two interviewees.

## Discussion

The results of this mixed-methods study provide insight into the current areas of strengths and potential improvements in that general surgery training programs in Saudi Arabia, which can resonate with other general surgery training programs. it is crucial to recognize that a low level of satisfaction with the training program's EE can have a detrimental effect on the wellbeing of trainees. Hence, it is pertinent to understand the underpinnings leading to low satisfaction and work towards improving it [16].

Over the past several years, few studies have attempted to examine various elements of the EE and trainees' satisfaction in GS training program in Saudi Arabia [10]. Acknowledging that postgraduate training program are undergoing quality improvements by the SCFHS, potentially affecting the representativeness of prior data to the current situation. In 2009, a study was conducted to assess educational climate in surgical programs in Riyadh, through measuring the satisfaction rate of surgical residents. This study demonstrated that approximately 78 % were not satisfied with their surgical programs and 85 % with their operative experiences [8]. Similarly, in 2016, a study was done in King Faisal Specialist Hospital and Research Center in Jeddah showed that all participants, regardless of their level, expressed a critical need for increasing the hands-on experience, and at least 65 % reported low or no operation room exposure [9]. Comparatively, our current study demonstrates some improvements in the aforementioned aspects. Nonetheless, the levels of satisfaction remain subpar, indicating the necessity for further efforts to enhance the program.

A major factor leading to an overall state of dissatisfaction, potential attrition, as well as symptoms of burnout, seem to be related to the quality of operative teaching. These findings are not particularly surprising [17]. Lee et al. (2017) found that supporting surgical residents academically improved their resilience and well-being. During the interviews, we found that residents that report meaningful learning experiences through simulation-based learning and practice, tended to have higher levels of overall satisfaction and report playing effective intra-operative roles, while having comparative experiences otherwise with those who were unsatisfied. Hence, our study findings emphasize on the need for focused efforts to improve the academic environment and teaching quality in surgical training programs. This element seems to play a principal role in the trainees' overall experience and potentially affect their wellbeing as well. This is a call to encourage medical educators' participation and fostering simulation-based training in the surgery programs.

The dissatisfaction with research opportunities and mentorship is consistent with previous studies evaluating the surgical training experience in Saudi Arabia [8]. Aldossary et al. (2020) depicts how the structure of clinical rotations, absence of mentoring research staff, and the lack of funding act as major barriers to foster the research environment among general surgery residents in Saudi Arabia [18]. This study's findings align with these results, highlighting the need for significant improvements in research opportunities and mentorship programs.

The study also found that nearly half of the residents suffer symptoms of burn-out and considered changing the general surgery program, with lifestyle and work environment being the primary reasons. This finding is consistent with studies conducted that have identified work-life balance and lifestyle issues as significant sources of stress and burnout in surgical residents. In fact, the rates of burnout identified in this study seem to be slightly lower than those identified in local and international literature [19]. Nonetheless, interventions are needed to improve work-life balance and optimize the workload on surgical residents, such as the ongoing implementation of duty-hour restrictions and providing counselling and support for personal and family responsibilities to promote wellbeing [20].

When comparing our results to international studies, we found both similarities and differences. For example, in the United States (US), the percentage of residents who considered quitting general surgery residency is comparable to our findings [21]. Regarding burnout, we found similarly high rates across all three aspects (emotional exhaustion, depersonalization, and personal accomplishment) when compared to US results [21]. Likewise, a study in Pakistan reported that 79 % of general surgery residents experienced high burnout rates [22]. In contrast, a study in Spain found that less than half (47.6 %) of the residents reported burnout symptoms [23]. In terms of satisfaction, our results showed that half of the residents were not satisfied with due to factors related to training, which differs from the US, where 85 % of general surgery residents reported being satisfied with their training [24]. These comparisons suggest that while there are some shared challenges faced by general surgery residents globally, the degree of these issues may vary across different healthcare systems and cultural contexts. Hence, the addition of an explanatory interviews - besides that statistical data - in this study helps in clearly elucidating these challenges that can then be tackled.

While this study highlights the importance of supervised learning activities and operative teaching as leading factors effecting GS trainees' satisfaction, it has several limitations to consider. These limitations include self-selection bias and a limited sample size. The study only included general surgery residents in Saudi Arabia, limiting the generalizability of its findings. Additionally, the study did not consider factors outside the EE, such as personal and social circumstances, that may affect residents' wellbeing. Finally, this study did not assess the impact of improving the EE on residents' wellbeing, which could be a topic for future research.

## Conclusion

This mixed-methods study provides evidence that general surgery trainees in Saudi Arabia are not optimally satisfied with their training program's EE, leading to negative impacts on their well-being. Despite satisfactory exposure to surgical cases the study identified several key factors contributing to dissatisfaction, including suboptimal educational delivery, inadequate operative teaching, and a lack of senior supervision and feedback.

To address these concerns, the study recommends enhancing the educational experience (EE) by incorporating simulation-based training to improve technical proficiency and boost trainees' confidence. Additionally, implementing effective mentorship programs can provide valuable support for personal and professional development, offering career guidance and assistance. Furthermore, it is crucial to establish support mechanisms to improve residents' overall well-being.

## Key message

This study reveals dissatisfaction and high rates of burnout among general surgery trainees in Saudi Arabia. Recommendations include implementing simulation training and mentorship programs to enhance the educational environment and support trainee wellbeing.

## CRediT authorship contribution statement

**Mohammed F. Shaheen:** Writing – review & editing, Writing – original draft, Visualization, Validation, Supervision, Software, Resources, Project administration, Methodology, Investigation, Formal analysis, Data curation, Conceptualization. **Abdulrahman Y. Alhabeeb:** Writing – review & editing, Writing – original draft, Visualization, Validation, Supervision, Software, Resources, Project administration, Methodology, Investigation, Formal analysis, Data curation. **Moustafa S. Alhamadh:** Writing – review & editing, Writing – original draft, Supervision, Software, Resources, Project administration, Investigation, Data curation. **Meshal A. Alothri:** Writing – review & editing, Writing – original draft, Investigation, Data curation. **Rakan S. Aldusari:** Writing – review & editing, Writing – original draft, Investigation, Data curation.

## Declaration of competing interest

All authors have explicitly stated that they have no conflicts of interest to declare.
